# Adsorption of Azo-Dye Orange II from Aqueous Solutions Using a Metal-Organic Framework Material: Iron- Benzenetricarboxylate 

**DOI:** 10.3390/ma7128037

**Published:** 2014-12-12

**Authors:** Elizabeth Rojas García, Ricardo López Medina, Marcos May Lozano, Isaías Hernández Pérez, Maria J. Valero, Ana M. Maubert Franco

**Affiliations:** 1 Departamento de Ciencias Básicas, Universidad Autónoma Metropolitana-Azcapotzalco, Av. San Pablo 180, Col. Reynosa Tamaulipas, México, D.F. 02200, Mexico; E-Mails: rlopez.ricardo@gmail.com (R.L.M.); mml@correo.azc.uam.mx (M.M.L.); ihp@correo.azc.uam.mx (I.H.P.); amf@correo.azc.uam.mx (A.M.M.F.); 2 Catalytic Spectroscopy Laboratory, Instituto de Catálisis y Petroleoquímica, Consejo Superior de Investigaciones Científicas (CSIC), Marie Curie 2, 28049 Madrid, Spain; E-Mail: mjvalero@icp.csic.es

**Keywords:** iron-benzenetricarboxylate, Orange II, azo-dye, adsorption process, Fe(BTC)

## Abstract

A Metal-Organic Framework (MOF), iron-benzenetricarboxylate (Fe(BTC)), has been studied for the adsorptive removal of azo-dye Orange II from aqueous solutions, where the effect of various parameters was tested and isotherm and kinetic models were suggested. The adsorption capacities of Fe(BTC) were much higher than those of an activated carbon. The experimental data can be best described by the Langmuir isotherm model (R^2^ > 0.997) and revealed the ability of Fe(BTC) to adsorb 435 mg of Orange II per gram of adsorbent at the optimal conditions. The kinetics of Orange II adsorption followed a pseudo-second-order kinetic model, indicating the coexistence of physisorption and chemisorption, with intra-particle diffusion being the rate controlling step. The thermodynamic study revealed that the adsorption of Orange II was feasible, spontaneous and exothermic process (−25.53 kJ·mol^−1^). The high recovery of the dye showed that Fe(BTC) can be employed as an effective and reusable adsorbent for the removal of Orange II from aqueous solutions and showed the economic interest of this adsorbent material for environmental purposes.

## 1. Introduction

In reality, nearly 40,000 dyes and pigments are color index listed, which consists of over 7000 different chemical structures [1]. Dyes are usually present in the effluent water of several industries, including textile, leather, paper, rubber, plastics, printing, cosmetics, pharmaceuticals and food industries. They contribute to water toxicity and represent an increasing danger for the environment, human and animals. Furthermore, dye-stuff wastes are known to be toxic [2], carcinogenic [3], mutagenic [4] and teratogenic [5]. Dyes are generally resistant to light, water, oxidizing agents and many chemicals and therefore difficult to degrade once released into the aquatic systems. Azo dyes are the largest and most versatile class of organic dye-stuffs. These contain one or more azo bonds (–N=N–) as a chromophore group in association with aromatic structures containing functional groups such as –OH and –SO_3_H. The complex aromatic structures of azo dyes make them more stable and more difficult to remove from the effluents discharged into the water bodies [6]. Thus, the removal of these dyes from wastewater is an important target from the environmental point of view.

Various conventional treatment methods for dye removal from wastewater include physical, chemical and biological processes such as, anaerobic treatment, trickling filters, flotation, chemical coagulation, electrochemical coagulation, membrane separation, advanced oxidation processes and photo-degradation, which have been studied so far [7,8]. However, the main disadvantages of these methods include the production of toxic sludge, high operational cost, technical limitations, lack of effective color reduction and sensitivity to a variable wastewater input. Therefore, the physical adsorption process at the solid-liquid interface is known to be a powerful method for removing contaminants owing to economical and environment-friendly reasons. The cost of an adsorption process mainly depends on the cost of the adsorbent and its regeneration. Table 1 shows the results obtained in recent research works, where the adsorption of Orange II onto various adsorbents has been studied [9,10,11,12,13,14,15,16,17,18,19,20,21,22]. Activated carbon is currently the most widely-used and -studied adsorbent. However, actually the Metal-Organic Frameworks (MOFs), as a new kind of adsorbent, have been applied for the adsorption of dyes [23]. 

Metal-organic frameworks (MOFs) [24] are crystalline porous materials that consist of metal-carboxylate units, metal ions and organic linkers, thereby the first term, “metal-organic”. These metal ions are coordinated to rigid organic molecules to form one-, two- or three-dimensional structures that can make the framework very porous. The particular interest in MOF materials is due to the easy tunability of their pore size and shape from a micro- to a meso-scale by changing the connectivity of the inorganic moiety and the nature of organic linkers. MOFs are well known for their various applications in gas store, separation, imaging, catalysis and drug delivery [25,26,27,28]. However, so far, there has been little report of the use of MOFs in the adsorptive removal of dyes; for example, adsorption of methyl orange from aqueous solutions over chromium-benzene-dicarboxylates and the adsorptive removal of methyl orange and methylene blue from contaminated water over iron terephthalate (MOF-235) [23,29]. However, no study on the adsorptive removal of azo-dyes using iron-benzenetricarboxylate (Fe(BTC)) metal-organic frameworks has been reported so far. Recently, Fe(BTC) has been used for aerobic oxidation of cycloalkenes and benzylic compounds [30], As(V) removal from aqueous solutions [31], “green” alcohol oxidations in water using aqueous H_2_O_2_ [32] and gas separation (CO_2_ and CH_4_) using mixed-matrix polymer membranes containing mesoporous Fe(BTC) [33]. 

**Table 1:** Comparison of the maximum monolayer adsorption capacities of azo-dye Orange II onto various adsorbent.

Adsorbent	T (K)	Q_0_ (mg·g^−1^)	Reference
Porous titania aerogel	323	420	[9]
Modified bentonite	306	239.5	[10]
Activated carbon (xerogels)	323	499.1	[11]
Activated carbon (fibers)	283	438	[12]
Activated carbon	303	384.3	[13]
Chitosan bead (cross-linked)	303	1940	[14]
Phosphoric acid-modified clam shell	286	1017.1	[15]
Amino-functionalized titanosilicate	298	189.1	[16]
Ammonia-tailored ordered mesoporous carbon	298	596	[17]
Carbon-alumina core-shell spheres	298	210	[18]
Nanoporous carbon from tomato waste	323	312.5	[19]
Canola stalks	298	25.6	[20]
Clay–alginate composites	298	980.5	[21]
Apricot shell activated carbon	298	13.98	[22]

**Scheme 1:**
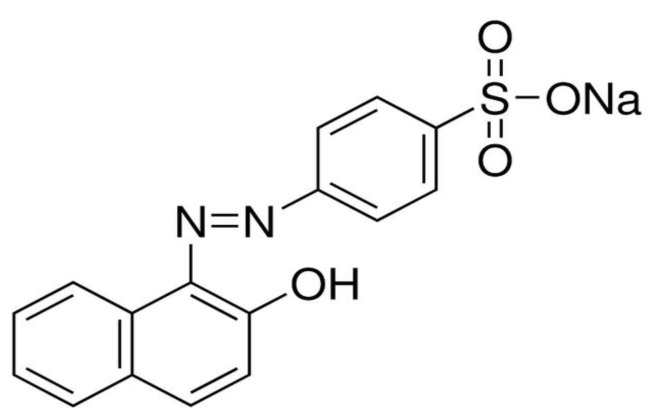
Molecular structure of azo-dye Orange II.

In this study, we report the kinetic and thermodynamic results of the adsorption of azo-dye Orange II from aqueous solution onto Fe(BTC). The molecular structure of azo-dye Orange II is shown in Scheme 1 and was used as a model molecule for this purpose. This dye has been selected, as it is inexpensive and widely used in textile, pulp and paper industries. The effects of various operating parameters, such as adsorbent amount, temperature, contact time and initial dye concentration, on Orange II dye removal were investigated. Then, the adsorption isotherms and kinetics of adsorption of Orange II were studied. In addition, the thermodynamic parameters were calculated for the Orange II adsorption on the adsorbent.

## 2. Results and Discussion

### 2.1. Materials Characterization

**Figure 1:**
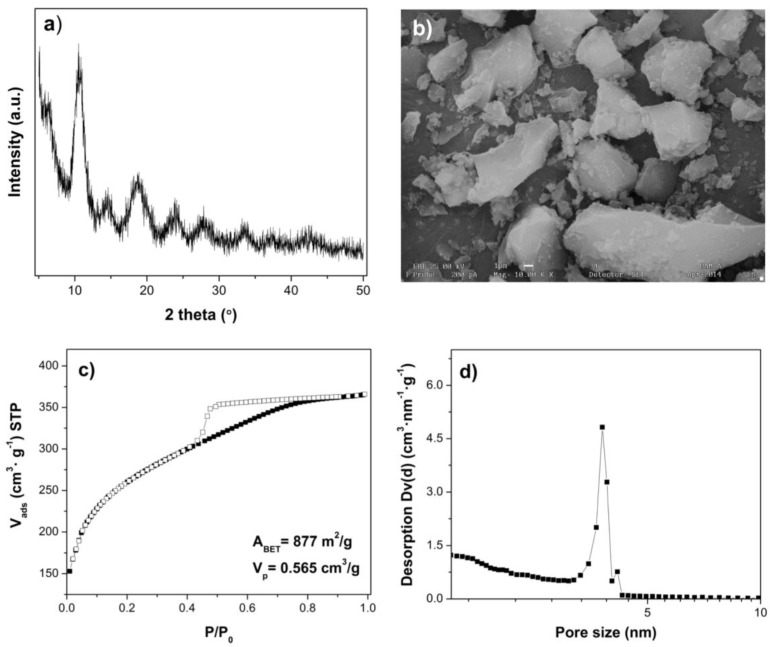
(**a**) Powder XRD pattern; (**b**) SEM image; (**c**) N_2_ adsorption isotherm and (**d**) pore size distribution for iron-benzenetricarboxylate (Fe(BTC)).

The X-ray diffraction pattern of the Fe(BTC) material shows several sharp and clearly-resolved reflections; these reflexes in the XRD profile provide evidence of the formation of a large scale structure, indicating the highly crystalline organization of the Fe(BTC) structure as shown in Figure 1a, which is in good agreement with previously reported studies [30,34,35]. The SEM image (Figure 1b) shows that the powders were irregular particles, with sizes on the micrometer scale. Figure 1c,d shows the N_2_ adsorption isotherm and the corresponding pore size distribution for Fe(BTC), which indicates that Fe(BTC) has a uniform porosity (average pore size = 3.5 nm), a BET specific surface area of 877 m^2^·g^−1^ and a total pore volume of 0.565 cm^3^·g^−1^. This is in accordance with the results reported in previous works [32,36] showing that high surface area and large pore volume potentiate that Fe(BTC) would be a good adsorbent material. Moreover, activated carbon shows a BET specific surface area of 974 m^2^·g^−1^ and total pore volume of 0.945 cm^3^·g^−1^. 

**Figure 2:**
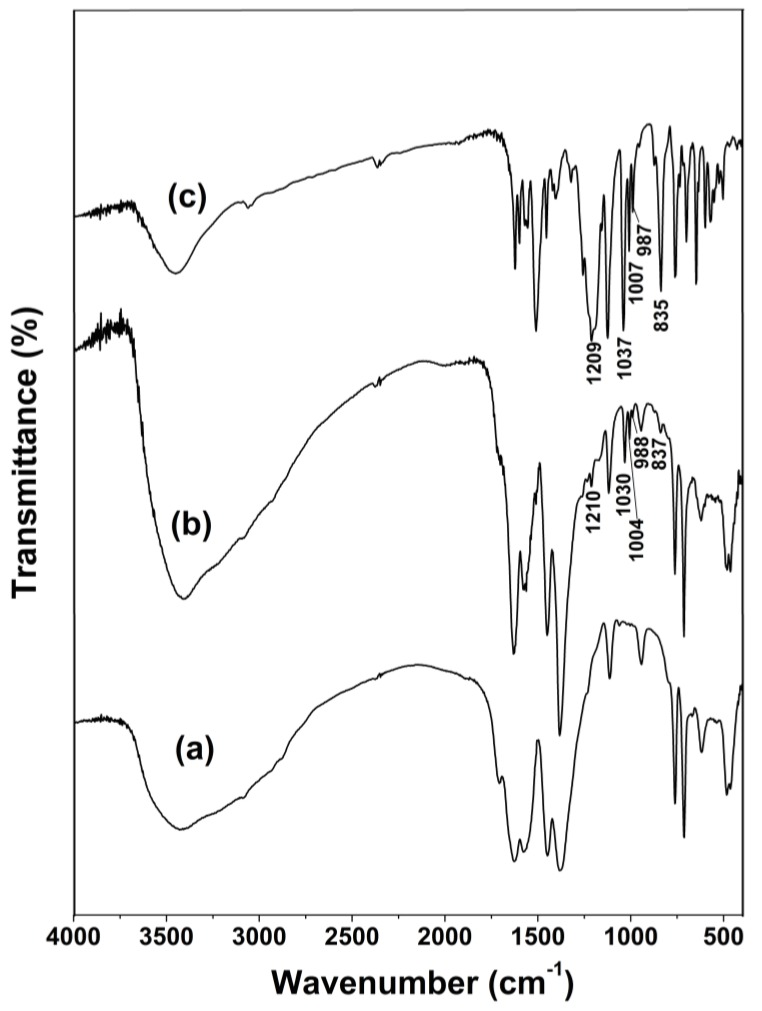
FTIR spectra of (**a**) Fe(BTC) material; (**b**) Fe(BTC) with adsorbed azo-dye Orange II dye and (**c**) azo-dye Orange II.

FTIR spectra of Fe(BTC), Fe(BTC) with adsorbed azo-dye Orange II and pure azo-dye Orange II are shown in Figure 2. The absorption bands from 3400 to 3650 cm^−1^ are due to the O–H stretching vibrations and the –OH bending vibrations of adsorbed water molecules. The infrared absorption spectrum of Fe(BTC) is very similar to those found in the literature and can be divided into two zones (Figure 2a) [34]. The first zone, below 1300 cm^−1^, shows various bands assigned to the vibrations of the BTC ligand, and the zone between 1300 and 1700 cm^−1^ is related to the carboxylate ligands and is thus indicative of the coordination of BTC to the iron sites. More precisely, the bands at 1626 and 1577 cm^−1^ and at 1448 and 1382 cm^−1^ correspond to the asymmetric and symmetric stretching vibrations of the carboxylate groups in BTC, respectively. The adsorption of the dye on the Fe(BTC) material is confirmed by FTIR spectroscopy which displays the characteristic bands of both Orange II dye [15] and Fe(BTC) material [34] (Figure 2b). In the FTIR spectrum of Orange II dye, the weak band at 1037 cm^−1^ corresponds to the –N=N– bond stretching vibration and the band at 1209 cm^−1^ is assigned to the –C–O– bond stretching vibration [16] (Figure 2c). The bands of the adsorbed Orange II dye on Fe(BTC) were similar to the bands of Orange II dye, but with a slight shift, indicating that the type of adsorption in the experiment was a physical adsorption (Figure 2b).

### 2.2. Effect of Adsorbent Concentration

**Figure 3:**
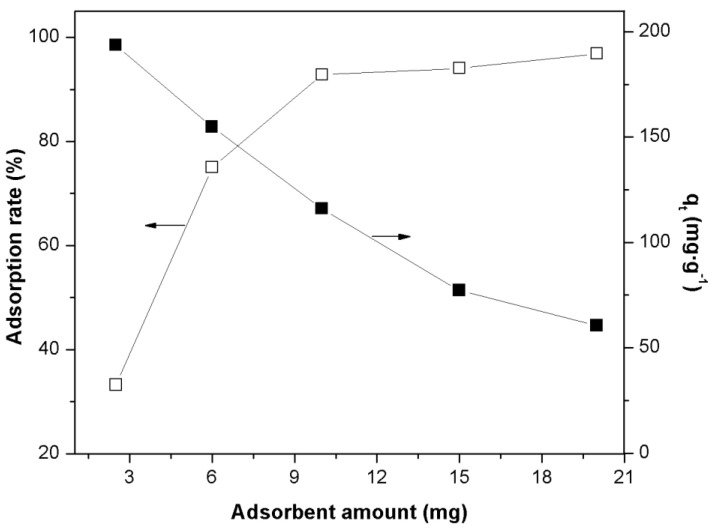
Effect of adsorbent amount on the adsorption rate (left y-axis) and adsorbed amount q_t_ (right y-axis) on the adsorption of azo-dye Orange II onto Fe(BTC) (initial dye concentration: 25 mg·L^−1^; contact time: 300 min; temperature: 298 K; pH 7).

Figure 3 shows the effect of adsorbent amount on the adsorption rate and adsorbed amount q_t_. It was observed that the adsorption rate increased with the increasing adsorbent amount until reaching an equilibrium value after 10 mg, which corresponds to 92% of the initial dye amount adsorbed onto Fe(BTC). An increase in the adsorption rate may be concluded due to the increase in more active functional groups, resulting in the availability of more adsorption sites. Under the constant dye concentration (25 mg·L^−1^) and volume (50 mL), we observed the reduction in the adsorbed amount q_t_ with an increase in the quantity of adsorbent. The reduction in the amount of dye adsorbed may be due to particle aggregation, resulting from high adsorbent mass [37]. This would lead to a decrease in total surface area of the adsorbent and an increase in diffusional path length. These observations are in agreement with other studies reported previously [9,38,39]. Therefore, in the following experiments, the adsorbent amount was fixed at 10 mg.

### 2.3. Effect of Initial Concentration of Orange II Dye 

**Figure 4:**
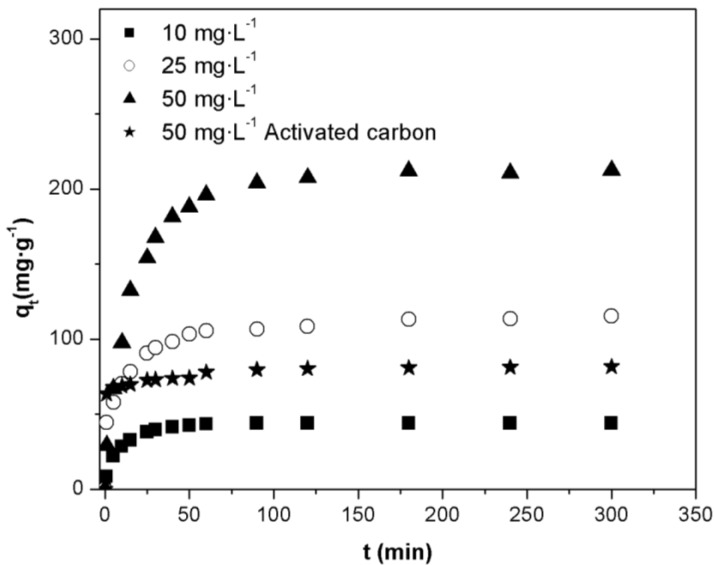
Effect of contact time and initial dye concentration on the adsorption of Orange II onto Fe(BTC) (adsorbent mass:10 mg; temperature: 308 K; pH 7).

Figure 4 represents the adsorbed amount q_t_ on Fe(BTC) and activated carbon *vs*. time for different initial dye concentrations (10, 25 and 50 mg·L^−1^). As shown in Figure 4, the adsorbed amount of Orange II onto Fe(BTC) is higher than that of the activated carbon at the same concentration (50 mg·L^−1^). Similar results were obtained at initial dye concentrations of 10 mg·g^−1^ and 25 mg·g^−1^ (see Supplementary materials Table S1). The adsorption of the dye at different concentrations is rapid in the initial stages and gradually decreases with the progress of adsorption, until equilibrium is reached between 80 min and 140 min. The adsorption was faster at the beginning, due to the higher availability of active sites on the Fe(BTC) surface. However, as time passes, these active sites were gradually occupied by the dye molecules and a decrease in the adsorptive sites for the residual dye molecules in the solution was observed. 

The results also showed that an increase in the initial dye concentration led to an increase in the adsorbed amount of dye onto Fe(BTC). The adsorbed amount of dye onto Fe(BTC) at equilibrium increased from 43.96 to 207.54 mg·g^−1^ with an increase in the initial Orange II concentrations from 10 to 50 mg·L^−1^. Similar observations have been reported for the adsorption of dyes on other adsorbents, such as adsorption of remazol black 5 onto biosorbent [20] and methyl orange and methylene blue onto iron terephthalate (MOF-235) [29]. 

### 2.4. Adsorption Isotherms

The distribution of dye molecule between the liquid phase and the adsorbent is a measure of the position of equilibrium in the adsorption process and can generally be expressed by one or more series of isotherm models. The adsorption isotherm models are fundamental for describing the interactive behavior between adsorbate and adsorbent and important for investigating mechanisms of adsorption. Isotherm data should accurately fit into different isotherm models to find the suitable model that can be used for adsorption process design [40]. In this study, Langmuir [41], Freundlich [42], Dubinin–Radushkevich [43] and Tempkin [44] isotherm models were used to describe the adsorption equilibrium data derived from the adsorption of Orange II over Fe(BTC) at different temperatures (298, 308 and 318 K). Equilibrium adsorption isotherms were studied with Orange II dye ranging from 10 to 250 mg·L^−1^ with a fixed adsorbent mass and pH.

#### 2.4.1. Langmuir Isotherm

**Figure 5:**
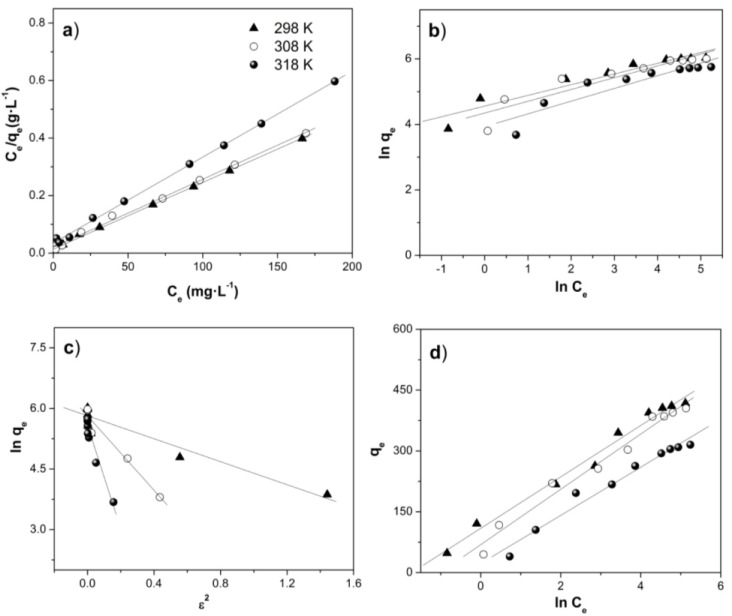
Adsorption isotherm models for Orange II dye adsorption over Fe(BTC): (**a**) Langmuir isotherm; (**b**) Freundlich isotherm; (**c**) D-R isotherm and (**d**) Tempkin isotherm.

The Langmuir isotherm model is based on several assumptions that include the following: (1) the surface is homogeneous; (2) adsorption is at a fixed number of definite, localized sites; (3) all sites are equivalent; (4) each site can accommodate only one adsorbate molecule; and (5) there is no interaction between adsorbed molecules, even on adjacent sites. The Langmuir adsorption isotherm has been successfully applied to many pollutant adsorption processes from aqueous solution [37,38]. The linear form of the Langmuir model is represented as follows [38,41]:

(1) $$\frac{{\text{C}}_{\text{e}}}{{\text{q}}_{\text{e}}}=\frac{{\text{C}}_{\text{e}}}{{\text{Q}}_{0}}+\frac{1}{{\text{bQ}}_{0}\;}$$

where, C_e_ is the liquid-phase concentrations of dye at equilibrium (mg·L^−1^), q_e_ the amount of dye adsorbed at equilibrium (mg·g^−1^), Q_0_ the maximum monolayer adsorption capacity (mg·g^−1^), and b the Langmuir constant (L·mg^−1^ or L·mol^−1^). A linear plot of (C_e_/q_e_) *vs*. C_e_ is obtained from the Langmuir model, as shown in Figure 5a. The values of Q_0_ and b were calculated from the slope and intercept of the different straight lines representing to different temperatures (Table 2). All of the correlation coefficients, R^2^, for the isotherms were higher than 0.997 at the three temperatures, indicating that the adsorption of Orange II over Fe(BTC) can be adequately described by the Langmuir isotherm model. The conformity of the experimental data with the Langmuir model was in agreement with most of the previously published experiments for other researches [9,15]. 

The maximum monolayer adsorption capacity of Fe(BTC) decreases with increasing of the temperature, which were 435 mg·g^−1^ at 298 K, 417 mg·g^−1^ at 308 K and 333 mg·g^−1^ at 318 K (Table 2). Moreover, the maximum monolayer adsorption capacity of Orange II over Fe(BTC) is larger than that of the activated carbon by around five-times at the same temperature (298 K) (Table 2). Both adsorbents show very similar textural properties, however, Fe(BTC) proved to be a more effective adsorbent for Orange II removal from aqueous solution. It could also be deduced that the electrostatic repulsive forces between the carbon surface and dye molecules might also contribute to resisting the adsorption of Orange II and result in lower adsorption capacity [17].

From the value of b deduced from the Langmuir model, the equilibrium parameter (R_L_) was calculated using the following equation [8,45]:

(2) $${\text{R}}_{\text{L}}=\frac{1}{1+{\text{bC}}_{0}}$$

where, C_0_ is the initial dye concentration. The value of R_L_ indicates whether the isotherm is unfavorable (R_L_ > 1), linear (R_L_ = 1), favorable (0 < R_L_ < 1) or irreversible (R_L_ = 0) [8]. The R_L_ values for Orange II adsorption over Fe(BTC) were less than one and greater than zero, showing a favorable adsorption (Table 2).

**Table 2:** Langmuir, Freundlich, Dubinin–Radushkevich (D-R) and Tempkin adsorption isotherms for Orange II adsorption onto Fe(BTC) and activated carbon at different temperatures.

T (K)	Langmuir isotherm				Freundlich isotherm			D-R isotherm			Tempkin isotherm		
	Q_0_ (mg·g^−1^)	b (L·mg^−1^)	R_L_	R^2^	n	K_F_ (mg·g^−1^)	R^2^	E (kJ·mol^−1^)	Q_D-R_ (mg·g^−1^)	R^2^	b_T_	K_T_ (L·mg^−1^)	R^2^
298 *	85	0.3656	0.011–0.215	0.999	5.59	41.10	0.968	1.469	76.13	0.804	246.16	54.538	0.953
298	435	0.1643	0.024–0.378	0.998	3.06	95.61	0.914	1.469	336.46	0.899	39.06	5.596	0.985
308	417	0.1154	0.033–0.148	0.997	2.78	77.41	0.857	1.519	335.89	0.937	37.55	2.754	0.977
318	333	0.0860	0.044–0.538	0.999	2.57	51.05	0.840	1.568	265.79	0.937	44.50	1.456	0.971

* Activated carbon.

#### 2.4.2. Freundlich Isotherm

The Freundlich isotherm model describes the adsorption of solutes from a liquid to a solid surface and assumes that different sites with several adsorption energies are involved. It gives a representation of the adsorption equilibrium between an adsorbent in solution and the surface of the adsorbent, using a multi-site adsorption isotherm for heterogeneous surfaces. The Freundlich model can be represented by the linear form as follows [18,19,42]:

(3) $$\text{ln}{\text{q}}_{\text{e}}=\text{ln}{\text{K}}_{\text{F}}\;+\left(\frac{1}{\text{n}}\right)\text{ln}{\text{C}}_{\text{e}}$$

where, C_e_ is the liquid-phase concentrations of dye at equilibrium (mg·L^−1^) and q_e_ the amount of dye adsorbed at equilibrium (mg·g^−1^). K_F_ and 1/n are Freundlich constants, where n indicates the degree of to which an adsorption process is favorable and K_F_ (mg·g^−1^) (L·mg^−1^)^1/n^ is the adsorption capacity of the adsorbent. K_F_ and 1/n can be determined from the linear plot of ln (q_e_) *vs*. ln (C_e_) (Figure 5b). The Freundlich constants and correlation coefficients at different temperatures are listed in Table 2. In general, as the K_F_ increases the adsorption capacity of the adsorbent increases. If *n* < 1, this means poor adsorption; from one to two means moderately difficult adsorption; and from two to ten good adsorption [46]. All of the n values obtained from the Freundlich model are more than unity, indicating that adsorption of Orange II dye on the Fe(BTC) and activated carbon is favorable (Table 2). Its correlation coefficients (0.840 < R^2^ < 0.968) are much lower than those for the Langmuir isotherm, suggesting that the Langmuir isotherm model is the best model to fit the experimental data. 

#### 2.4.3. Dubinin–Radushkevich Isotherm 

The nature of adsorption (physical or chemical) was also analyzed by the Dubinin–Radushkevich (D-R) isotherm. The D-R isotherm model is generally applied to express the adsorption mechanism with a Gaussian energy distribution onto a heterogeneous surface. The D–R isotherm model can be expressed as [47]:

(4) $${\text{lnq}}_{\text{e}}={\text{lnQ}}_{\text{D}-\text{R}}-{\text{βε}}^{2}$$

where, q_e_ is the amount of dye adsorbed at equilibrium (mg·g^−1^), Q_D-R_ the theoretical adsorption capacity (mg·g^−1^), β is the constant of the sorption energy and ε is Polanyi potential, which is expressed by the following equation [47]:

(5) $$\text{ε}=\text{ RTln}\left(1+\frac{1}{{\text{C}}_{\text{e}}}\right)$$

where, C_e_ is the liquid-phase concentrations of dye at equilibrium (mg·L^−1^), R the ideal gas constant (8.314 × 10^−3^ kJ·K^−1^·mol^−1^) and T the absolute temperature (K).

The energy of adsorption is the free energy of the transfer of 1 mol of solute from infinity (in solution) to the surface of the adsorbent. The mean value of adsorption energy, E, can be calculated from the constant of the sorption energy (D-R pattern) as follows [47]:

(6) $$\text{E}=\frac{1}{\sqrt{2\text{β}}}$$

The magnitude of E (kJ·mol^−1^) is used for estimating the type of adsorption mechanism. If this value is between 8 and 16 kJ·mol^−1^, the adsorption process is controlled by a chemical mechanism, while for E < 8 kJ·mol^−1^, the adsorption process proceeds through a physical mechanism [48]. The calculated values of E (Table 2 and Figure 5c) suggested that the adsorption of dye occurs via physical adsorption. Kousha *et al*. [48] also have showen that the value obtained for the adsorption energy (0.29 kJ·mol^−1^) of acid Orange II dye into macroalga *Stoechospermum marginatum* results from a physisorption process. 

#### 2.4.4. Tempkin Isotherm 

The Tempkin isotherm model takes into account the interactions between adsorbent and dye to be adsorbed and is based on the assumption that the free energy of adsorption is a function of the surface coverage. The linear form of the Tempkin isotherm model is represented as follows (Equation (7)) [45,49]:

(7) $${\text{q}}_{\text{e}}=\left(\frac{\text{RT}}{{\text{b}}_{\text{T}}}\right){\text{lnK}}_{\text{T}}+\left(\frac{\text{RT}}{{\text{b}}_{\text{T}}}\right){\text{lnC}}_{\text{e}}$$

where K_T_ is the Temkin isotherm constant (L·g^−1^), b_T_ the constant related to the heat of adsorption (kJ·mol^−1^), R the ideal gas constant (8.314 × 10^−3^ kJ·K^−1^·mol^−1^) and T the absolute temperature (K). K_T_ and b_T_ can be determined from the linear plot of q_e_ *vs*. ln (C_e_) (Figure 5d). The characteristic parameters of the Tempkin model at several temperatures, as well as the correlation coefficients, R^2^, are listed in Table 2. It was observed that the Tempkin model did not show a good fit to the data compared with the Langmuir model.

The results revealed that the R^2^ of the Langmuir isotherm was greater than those of the other models, indicating that the Langmuir isotherm better represented the adsorption of Orange II over Fe(BTC). Therefore, the Langmuir model confirms that the adsorption is monolayer with a homogenous distribution of adsorption sites.

### 2.5. Adsorption Kinetics

**Figure 6:**
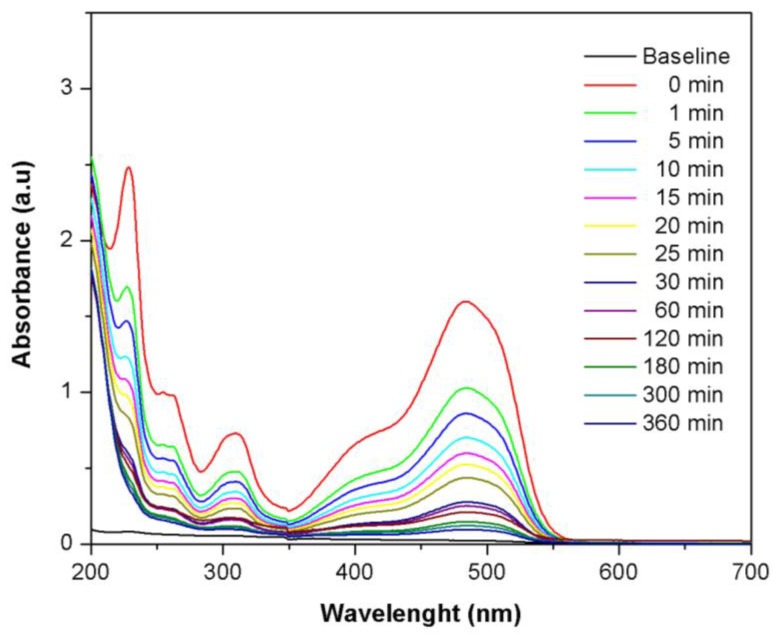
UV-Vis spectra of the adsorption of Orange II dye over Fe(BTC) at different times (initial concentration: 25 mg·L^−1^; adsorbent mass: 10 mg; temperature: 308 K; pH 7).

UV-Vis spectra of the adsorption of Orange II over Fe(BTC) at different time show that the adsorption process could be divided into two stages: (1) a rapid adsorption of dye on the adsorbent surface occurs at the initial stage (0 to 15 min); and (2) then, a slow adsorption of dye occurred after these times until equilibrium was reached (Figure 6). The formation of new compounds was not detected in the UV-Vis spectra. This indicates that decolorization mainly results from the process of adsorption and not from Orange II degradation. Similar results have been reported for the adsorption of dyes on other adsorbents [50].

**Figure 7:**
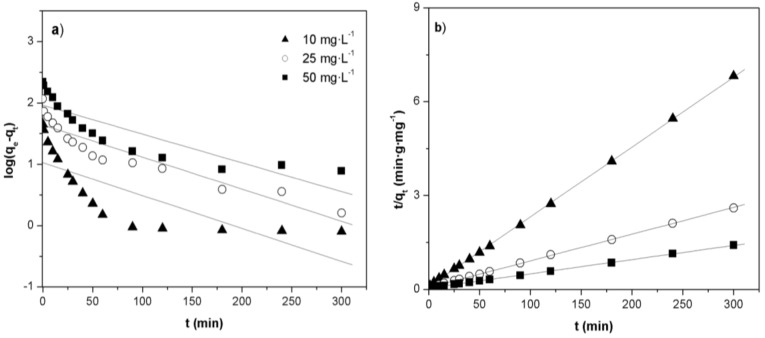
Kinetic models plots for the adsorption of Orange II on Fe(BTC) at different initial concentrations: (**a**) pseudo-first-order kinetic model plot; (**b**) pseudo-second-order kinetic model plot (adsorbent mass: 10 mg; temperature: 308 K; pH 7).

The adsorption kinetics is an important parameter for designing adsorption systems and is required for selecting the optimum operating conditions for a pilot-scale process. In order to investigate the adsorption kinetics of Orange II dye over Fe(BTC), two models have been studied: the pseudo-first-order kinetic model and pseudo-second order kinetic model. The linear form of the pseudo-first-order equation is given as follows [39]:

(8) $$\text{log}\left({\text{q}}_{\text{e}}-{\text{q}}_{\text{t}}\right)=\text{log}q_{e}-\frac{k_{1}}{2.303}t$$

where q_e_ and q_t_ are the amounts of dye adsorbed (mg·g^−1^) at equilibrium and at time t (min), respectively, and k_1_ is the rate constant of pseudo-first-order kinetics (min^−1^). Values of k_1_ were calculated from the plots of log (q_e_−q_t_) *vs*. time (Table 3 and Figure 7a). From Table 3, the lineal regression coefficients obtained from the pseudo-first-order kinetic model were found to be low. Furthermore, there were significant differences between the calculated and experimental q_e_ values, indicating that the first order model does not reproduce the adsorption kinetics of Orange II over Fe(BTC).

**Table 3:** Kinetic parameters for the adsorption of Orange II over Fe(BTC) at different concentrations (adsorbent mass: 10 mg; temperature: 308 K; pH 7).

C_0_ (mg·g^−1^)	q_e,exp_ (mg·g^−1^)	Pseudo-first-order			Pseudo-second-order		
		q_e,cal_ (mg·g^−1^)	k_1_ (min^−1^)	R^2^	q_e,cal_ (mg·g^−1^)	k_2_ (g·mg·^−1^·min^−1^)	R^2^
10	43.98	10.67	1.24 × 10^−2^	0.611	44.64	4.14 × 10^−3^	0.999
25	115.49	45.91	1.31 × 10^−2^	0.820	117.09	1.46 × 10^−3^	0.999
50	212.49	91.94	1.08 × 10^−2^	0.750	220.26	5.64 × 10^−4^	0.999

The experimental data were also examined by the pseudo-second-order kinetic model which is given by the following equation [39,51]:

(9) $$\frac{\text{t}}{{\text{q}}_{\text{t}}}=\frac{1}{{\text{k}}_{2}{\text{q}}_{\text{e}}^{2}}+\frac{1}{{\text{q}}_{\text{e}}}\text{t}$$

where k_2_ is the rate constant of pseudo-second-order kinetics (g·mg^−1^min^−1^). The rate constant, the amounts of dye adsorbed and the R^2^ values are showed in Table 3. The plots of t/q_t_ *vs*. time at different initial dye concentrations show excellent linearity (Figure 7b). The results show that the pseudo second-order kinetic model fits the experimental data better with linear regression coefficients of 0.999 (R^2^ > 0.999) at all initial dye concentrations. Similar results were observed at the different temperatures tested (298 and 318 K) (see Supplementary materials Figures S1 and S2 and Table S2). Furthermore, the calculated q_e_ values for the pseudo-second-order kinetic model show good agreement with the experimental q_e_ values. From Table 3, the rate constant k_2_ decreased with the increasing dye initial concentration, indicating that the chemisorption was significant. The chemisorption might be the rate limiting step where valency forces are involved via electrons sharing or exchange between the adsorbent and the adsorbate [51]. 

### 2.6. Thermodynamic Analysis

**Figure 8:**
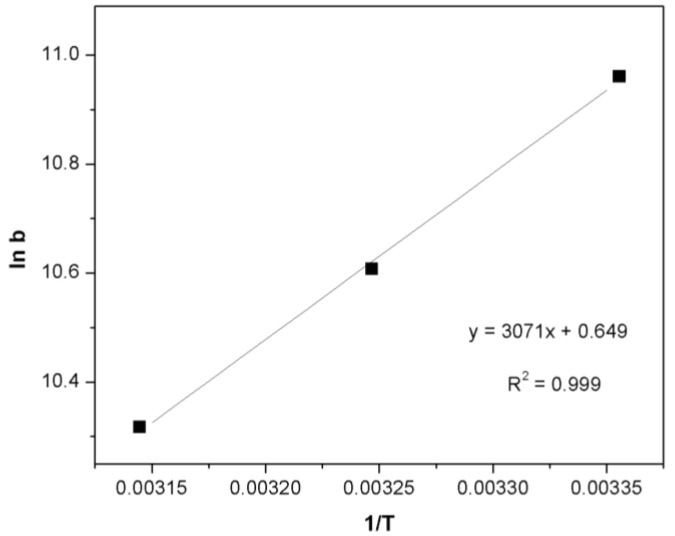
Van’t Hoff plots to get the ΔH° and ΔS° of the Orange II adsorption over Fe(BTC).

The thermodynamic parameters that must be considered to determine the process are Gibbs free energy change (ΔG°) (Equation (10)), enthalpy of adsorption (ΔH°) and entropy change (ΔS°) (Equation (11)) due to the transfer of a unit mole of solute from solution on the solid-liquid interface. These parameters were calculated using the following equations:

(10) ΔG=−RTlnb

(11) $$\text{lnb}=\;\frac{\Delta {\text{S}}^{\circ }}{\text{R}}-\frac{\Delta {\text{H}}^{\circ }}{\text{RT}}$$

where R is the ideal gas constant (8.314 × 10^−3^ kJ·mol^−1^·K^−1^), b the Langmuir isotherm constant (L·mol^−1^), ΔG° the change in Gibbs free energy (kJ·mol^−1^), ΔH° the enthalpy of adsorption (kJ·mol^−1^) and ΔS° the entropy of adsorption (J·mol^−1^·K^−1^). The results of the thermodynamic calculations are shown in Table 4. The pattern b can be obtained from the slope/intercept of the Langmuir plot of Figure 5a. The negative values of change in Gibbs free energy (ΔG°) shown in Table 4 indicated the feasibility of the process and the spontaneous nature of the adsorption under the experimental conditions used. ΔH° and ΔS° can be determined from the slope and the intercept of the linear plot of ln b *vs*. 1/T, as shown in Figure 8, and the results are also shown in Table 4. Kara *et al*. [52] suggested that the process is considered as physisorption when the ΔH° values are less than 40 kJ·mol^−1^. The negative value for enthalpy change ΔH° (−25.53 kJ·mol^−1^), confirms that the adsorption of Orange II over Fe(BTC) is an exothermic process. The negative value of ΔS° (−5.40 J·mol^−1^·K^−1^) reflects the decrease in the disorder of the system at the solid-solution interface, and no significant change occurred in the internal structure of the adsorbent during the adsorption process.

**Table 4:** Thermodynamic parameters of Orange II adsorption over Fe(BTC) at different temperatures.

Temperature (K)	ln b (L·mol^−1^)	ΔG° (kJ·mol^−1^)	ΔH° (kJ·mol^−1^)	ΔS° (J·mol^−1^·K^−1^)
298	10.96	−27.16	−25.53	−5.40
308	10.60	−27.16	-	-
318	10.31	−27.26	-	-

### 2.7. Adsorption Mechanism

**Figure 9:**
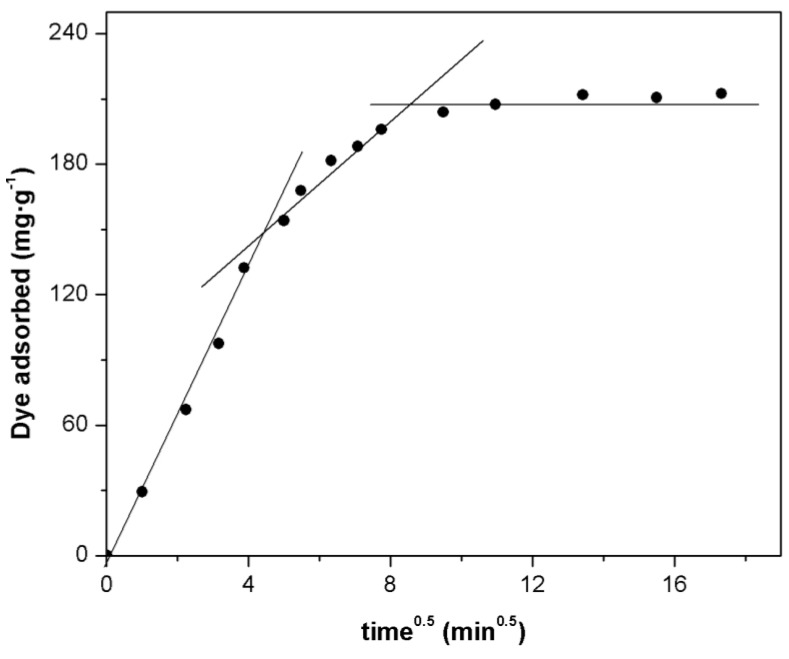
Intra-particle diffusion model plot of dye adsorbed over Fe(BTC). (Initial concentration: 50 mg·L^−1^; adsorbent mass: 10 mg; temperature: 308 K and pH 7).

Generally, the intra-particle diffusion model (IPD) is used to identify the mechanism involved in the adsorption process. This model assumes that intra-particle diffusion is the rate-controlling step, which is generally the case for well-mixed solutions. The intra-particle diffusion model is a single-resistance model in nature and might be described by Weber and Morris, which is based on Fick’s second law [53], where it is indicated that if the adsorption process is influenced by the intra-particle diffusion, the adsorbed amount q_t_ should vary linearly with the square root of time. The intra-particle diffusion model can be described by the following equation [48]:

(12) $${\text{q}}_{\text{t}}={\text{k}}_{\text{p}}{\text{t}}^{0.5}+\text{C}$$

where, k_P_ is the intra-particle diffusion rate constant (mg·g^−1^·min^−0.5^) and C is the intra-particle diffusion constant. The k_p_ value was calculated from the slope of the straight line part of the curve (q_t_ *vs*. t^0.5^). Figure 9 shows the plot of the data obtained for the adsorption of Orange II onto Fe(BTC) at 308 K. This figure revealed that the plot is not linear over the whole time range; however, it exhibit a tri-linearity, revealing the existence of three successive adsorption stages of mass transport with a decreasing rate, which also have been observed by previous investigations [14,36]. Similar results were obtained at 298 and 318 K (see Supplementary materials Figures S3 and S4). Abramian *et al*. [9] showed that this tri-linearity can be attributed to: (1) the external surface adsorption correlated to the boundary layer diffusion; (2) the intra-particle diffusion states where this step is highly involved in the rate control of this mechanism; and (3) the final equilibrium stage, where the intra-particle diffusion starts to slow down due to the low dye concentration in the solution. It is important to note the fact that when the second linear plots do not pass through the origin, this is indicative of some degree of boundary layer control and further shows that the intra-particle diffusion is not the only rate controlling step, but some other processes may also control the rate of adsorption [9,23]. 

### 2.8. Regeneration of Adsorbent 

**Figure 10:**
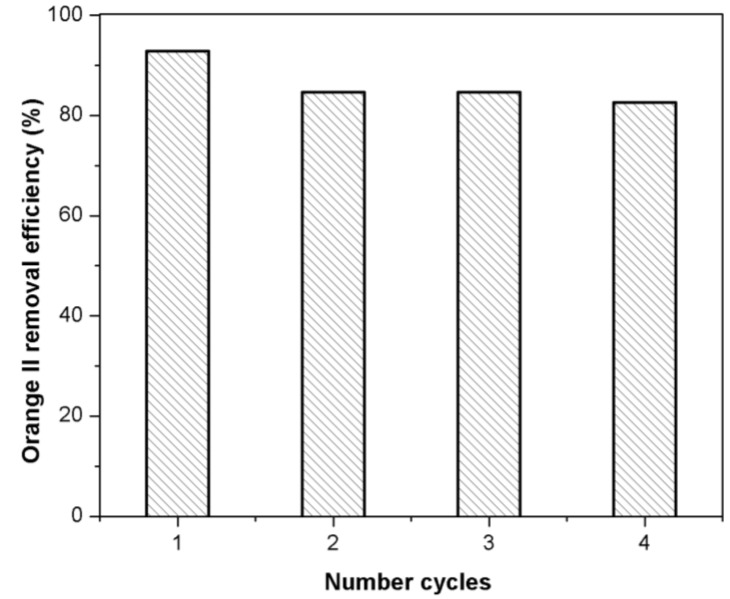
Orange II removal of the recycled Fe(BTC) (initial concentration: 25 mg·L^−1^; adsorbent mass: 10 mg; temperature: 298 K; pH 7).

The facile regeneration and reusability test of Fe(BTC) is quite important for an industrial application (Figure 10). The adsorption capacity of the regenerated Fe(BTC) (second, third and fourth runs) was tested under similar conditions as in Section 3.3 and compared to the first use (first run) (Figure 10). This shows that the adsorption capacity of Orange II onto Fe(BTC) decreases slowly with increasing cycle number. After of four cycles, the Orange II removal efficiency is still above 83%. Furthermore, Fe(BTC) can be easily regenerated and re-used several times, indicating that this adsorbent could have great potential for the dye adsorption of wastewater. 

## 3. Experimental Section

### 3.1. Synthesis of Fe(BTC) Material

All reagents and starting materials were obtained commercially from Sigma-Aldrich, and were used as received without any further purification. Fe(BTC) was prepared adapting the procedure reported by Petit *et al*. [54]. An exact amount of iron (III) nitrate nonahydrated (3.52 g, 8.7 mmol) and benzene-1,3,5-tricarboxylic acid (H_3_BTC) (1.76 g, 8.3 mmol) were mixed and suspended in 30 mL of N,N dimethylformamide followed by stirring and sonication for 5 min. After, 30 mL of ethanol were added to the mixture, which was then stirred in an ultrasonic bath for 5 min. Subsequently, 30 mL of deionized water were added to the mixture and stirred in an ultrasonic bath for 30 min in order to allow the complete dissolution of all components. The mixture was then heated in a sand bath and kept at 358 K for 24 h under constant stirring. After cooling, the brown crystals were filtered, washed and immersed in methanol. After 24 h the methanol was removed by filtration and changed with fresh methanol. This procedure was repeated three times. Finally, the crystals were dried at 423 K for 24 h. Activated carbon was obtained commercially from Merck chemical company and was used as received without any treatments and was used as a reference adsorbent. 

### 3.2. Characterization 

Powder XRD patterns were recorded on a Philips X’Pert diffractometer (Almelo, The Netherlands) using CuK_α_ radiation (λ = 0.154178 nm) as the incident beam. The specimens were scanned in the range of 4–80° (2θ) with a scan rate of 0.02° s^−1^. Nitrogen adsorption isotherms were recorded with an automatic Micromeritics ASAP-2000 apparatus (Norcross, GA, USA). Prior to the adsorption experiments, samples were outgassed at 413 K for 2 h. BET areas were computed from the adsorption isotherms (0.05 < P/P_0_ < 0.27), taking a value of 0.164 nm^2^ for the cross-section of the adsorbed N_2_ molecule at 77 K. Fourier Transform Infrared (FTIR) spectra were obtained on a Thermo Scientific Nicolet 750 FTIR spectrometer (Champaign, IL, USA) using the transmission KBr pellet technique, operating in the range of 4000 to 400 cm^−1^, and were recorded with a resolution of 4 cm^−1^. After the recording, 64 interferograms were collected. The crystallite morphologies were examined using a Scanning Electron Microscope (SEM, LEO 440, Cambridge, UK) operated at 25 kV.

### 3.3. Adsorption Tests

Before adsorption, the adsorbent was dried overnight at 373 K and kept in a desiccator. A known quantity of adsorbent was added to an aqueous solution of Orange II (50 mL), with an initial concentration in the range of 10 to 250 mg·L^−1^, with continuous magnetic stirring at different temperatures (298, 308 and 318 K). The concentrations of the organic dye at different adsorption times were determined by UV-Vis spectrophotometry (Varian, Cary 1G) by measuring the maximum absorbance of the solution, located at λ = 485 nm, and using a calibration function of concentration-absorbance previously determined [16,48]. For each experimental run, the adsorption process was carried out until equilibrium, *i.e.*, when there was no appreciable change in concentration. After adsorption, the supernatants were separated from the adsorbent by centrifugation (15,000 rpm, 5 min), and the dye concentration of the obtained supernatants was determined. The used adsorbent was regenerated by washing it with ethanol (usually 0.05 g with 10 mL) for 30 min at room temperature, after that the adsorbent was filtered and dried at 423 K for 24 h.

The amount of dye adsorbed at time t was calculated from the following equation [7,16,27]:

(13) $${\text{q}}_{\text{t}}=\frac{\left({\text{C}}_{\text{i}}-{\text{C}}_{\text{t}}\right)\text{V}}{\text{m}}$$

where, q_t_ is the amount of dye adsorbed at time t (mg·g^−1^), C_i_ the initial dye concentration in liquid phase (mg·L^−1^), C_t_ the liquid-phase dye concentration at time t (mg·L^−1^), V the volume of dye solution (L) and m the mass of the adsorbent (g). Finally, the adsorption rate was calculated using the following equation [48]:

(14) $$\text{Adsorption rate}\left(\text{\%}\right)=\frac{C_{i}-C_{t}}{C_{i}}\times 100$$

## 4. Conclusions

This paper presents the results of a detailed adsorption study for removing azo-dye Orange II from aqueous solutions over an MOF material (iron-benzenetricarboxylate). Operational parameters, such as the adsorbent mass, temperature, contact time and initial dye concentrations, clearly affect the removal efficiency. The results revealed that the R^2^ of the Langmuir isotherm model was greater than those of the other models, indicating that the Langmuir isotherm model better represented the adsorption of Orange II over Fe(BTC). In addition, the thermodynamic calculations indicated that the adsorption of Orange II over Fe(BTC) shows that the adsorption is feasible, spontaneous and exothermic process. Furthermore, no significant change occurred in the internal structure of the adsorbent during the adsorption process. Therefore, we conclude that Fe(BTC) material can be used as highly efficient adsorbents and reused for the removal of anionic azo-dyes from wastewater.

## Supplementary Materials

Supplementary materials can be accessed at: http://www.mdpi.com/1996-1944/7/12/8037/s1.

## References

[1] Demirbas A. (2009). Agricultural based activated carbons for the removal of dyes from aqueous solutions: A review. J. Hazard. Mater..

[2] Tsuboy M.S., Angeli J.P.F., Mantovani M.S., Knasmüller S., Umbuzeiro G.A., Ribeiro L.R. (2007). Genotoxic, mutagenic and cytotoxic effects of the commercial dye CI Disperse Blue 291 in the human hepatic cell line HepG2. Toxicol. in Vitro.

[3] Golka K., Kopps S., Myslak Z.W. (2004). Carcinogenicity of azo colorants: Influence of solubility and bioavailability. Toxicol. Lett..

[4] Chung K.T. (2000). Mutagenicity and carcinogenicity of aromatic amines metabolically produced from azo dyes. Environ. Carcino. Ecotox. Revs..

[5] Beaudoin A.R., Pickering M.J. (1960). Teratogenic activity of several synthetic compounds structurally related to trypan blue. Anat. Record.

[6] Crini G. (2006). Non-conventional low-cost adsorbents for dye removal: A review. Bioresour. Technol..

[7] Kornaros M., Lyberatos G. (2006). Biological treatment of wastewaters from a dye manufacturing company using a trickling filter. J. Hazard. Mater..

[8] Hall K., Eagleton L., Acrivos A., Vermeulen T. (1966). Pore-and solid-diffusion kinetics in fixed-bed adsorption under constant pattern conditions. Ind. Eng. Chem. Fundamen..

[9] Abramian L., El-Rassy H. (2009). Adsorption kinetics and thermodynamics of azo-dye Orange II onto highly porous titania aerogel. Chem. Eng. J..

[10] Ma J., Qi J., Yao C., Cui B., Zhang T., Li D. (2012). A novel bentonite-based adsorbent for anionic pollutant removal from water. Chem. Eng. J..

[11] Ribeiro R.S., Fathy N.A., Attia A.A., Silva A.M.T., Faria J.L., Gomes H.T. (2012). Activated carbon xerogels for the removal of the anionic azo dyes Orange II and Chromotrope 2R by adsorption and catalytic wet peroxide oxidation. Chem. Eng. J..

[12] Hsiu-Mei C., Ting-Chien C., San-De P., Hung-Lung C. (2009). Adsorption characteristics of Orange II and Chrysophenine on sludge adsorbent and activated carbon fibers. J. Hazard. Mater..

[13] Rodríguez A., García J., Ovejero G., Mestanza M. (2009). Adsorption of anionic and cationic dyes on activated carbon from aqueous solutions: Equilibrium and kinetics. J. Hazard. Mater..

[14] Chiou M.S., Ho P.Y., Li H.Y. (2004). Adsorption of anionic dyes in acid solutions using chemically cross-linked chitosan beads. Dyes Pigments.

[15] Ma J., Zou J., Cui B., Yao C., Li D. (2013). Adsorption of Orange II dye from aqueous solutions using phosphoric-acid modified clam shell powder. Desalin. Water Treat..

[16] Marçal L., de Faria E.H., Saltarelli M., Calefi P.S., Nassar E.J., Ciuffi K.J. (2011). Amine-functionalized titanosilicates prepared by the sol-gel process as adsorbents of the azo-dye Orange II. Ind. Eng. Chem. Res..

[17] He C., Hu X. (2011). Anionic dye adsorption on chemically modified ordered mesoporous carbons. Ind. Eng. Chem. Res..

[18] Zhou J., Tang C., Cheng B., Yu J., Jaroniec M. (2012). Rattle-type carbon−alumina core−shell spheres: Synthesis and application for adsorption of organic dyes. ACS Appl. Mater. Interfaces.

[19] Güzel F., Saygli H., Saygli G.A., Koyuncu F. (2014). Elimination of anionic dye by using nanoporous carbon prepared from an industrial biowaste. J. Mol. Liq..

[20] Hamzeh Y., Ashori A., Azadeh E., Abdulkhani A. (2012). Removal of acid Orange 7 and remazol black 5 reactive dyes from aqueous solutions using a novel biosorbent. Mater. Sci. Eng..

[21] Mandal S., Patil V.S., Mayadevi S. (2012). Alginate and hydrotalcite-like anionic clay composite systems: Synthesis, characterization and application studies. Micropor. Mesopor. Mat..

[22] Cao J., Wu Y., Jin Y., Yilihan P., Yang S. (2013). Dynamic adsorption of anionic dyes by apricot shell activated carbon. Desalin. Water Treat..

[23] Haque E., Lee J.E., Jang I.T., Hwang Y.K., Chang J.S., Jegal J., Jhung S.H. (2010). Adsorptive removal of methyl orange from aqueous solution with metal-organic frameworks, porous chromium-benzenedicarboxylates. J. Hazard. Mater..

[24] Férey G. (2008). Hybrid porous solids: Past, present, future. Chem. Soc. Rev..

[25] Millward A.R., Yaghi O.M. (2005). Metal-Organic Frameworks with exceptionally high capacity for storage of carbon dioxide at room temperature. J. Am. Chem. Soc..

[26] Farrusseng D., Aguado S., Pinel C. (2009). Metal–Organic Frameworks: Opportunities for catalysis. Angew. Chem. Int. Ed..

[27] Li S.L., Xu Q. (2013). Metal–organic frameworks as platforms for clean energy. Energy Environ. Sci..

[28] Li J.-R., Sculley J., Zhou H-C. (2012). Metal-Organic Frameworks for separations. Chem. Rev..

[29] Haque E., Jun J.W., Jhung S.H. (2011). Adsorptive removal of methyl orange and methylene blue from aqueous solution with a metal-organic framework material, iron terephthalate (MOF-235). J. Hazard. Mater..

[30] Opanasenko M., Dhakshinamoorthy A., Cêjka J., Garcia H. (2013). Deactivation pathways of the catalytic activity of metal-organic frameworks in condensation reactions. Chem. Cat. Chem..

[31] Zhu B.J., Yu X.Y., Jia Y., Peng F.M., Sun B., Zhang M.Y., Luo T., Liu J.H., Huang X.J. (2012). Iron and 1,3,5-benzenetricarboxylic metal−organic coordination polymers prepared by solvothermal method and their application in efficient As(V) removal from aqueous solutions. J. Phys. Chem. C.

[32] Hosseini-Monfared H., Näther C., Winkler H., Janiak C. (2012). Highly selective and “green” alcohol oxidations in water using aqueous 10% H_2_O_2_ and iron-benzenetricarboxylate metal–organic gel. Inorg. Chim. Acta.

[33] Shahid S., Nijmeijer K. (2014). High pressure gas separation performance of mixed-matrix poly mermembranes containing mesoporous Fe(BTC). J. Membr. Sci..

[34] Dhakshinamoorthy A., Alvaro M., Chevreau H., Horcajada P., Devic T., Serre C., Garcia H. (2012). Iron(III) metal–organic frameworks as solid Lewis acids for the isomerization of α-pinene oxide. Catal. Sci. Technol..

[35] Ploegmakers J., Japip S., Nijmeijer K. (2013). Mixed matrix membranes containing MOFs for ethylene/ethane separation Part A: Membrane preparation and characterization. J. Membrane Sci..

[36] Dhakshinamoorthy A., Alvaro M., Garcia H. (2012). Aerobic oxidation of cycloalkenes catalyzed by iron metal organic framework containing N-hydroxyphthalimide. J. Catal..

[37] Malekbala M.R., Hosseini S., Kazemi-Yazdi S., Masoudi Soltani S., Malekbala M.R. (2013). On the utilization of a lignocellulosic waste as an excellent dye remover: Modification, characterization and mechanism analysis. Chem. Eng. J..

[38] Wang T., Kailasam K., Xiao P., Chen G., Chen L., Wang L., Li J., Zhu J. (2014). Adsorption removal of organic dyes on covalent triazine framework (CTF). Micropor. Mesopor. Mater..

[39] Sarkar B., Xi Y., Megharaj M., Naidu R. (2011). Orange II adsorption on palygorskites modified with alkyl trimethylammonium and dialkyl dimethylammonium bromide—An isothermal and kinetic study. Appl. Clay Sci..

[40] Gil A., Assis F.C.C., Albeniz S., Korili S.A. (2011). Removal of dyes from wastewaters by adsorption on pillared clays. Chem. Eng. J..

[41] Langmuir I. (1916). The constitution and fundamental properties of solids and liquids. Part I. Solids. J. Am. Chem. Soc..

[42] Freundlich H.M.F. (1906). Over the adsorption in solution. J. Phys. Chem..

[43] Dubinin M.M., Zaverina E.D., Radushkevich L.V. (1947). Sorption and structure of active carbons. J. Phy. Chem..

[44] Tempkin M.I., Pyzhev V. (1939). Kinetics of ammonia synthesis on promoted iron catalyst. J. Phys. Chem. USSR.

[45] Günay A., Arslankaya E., Tosun I. (2007). Lead removal from aqueous solution by natural and pretreated clinoptilolite: Adsorption equilibrium and kinetics. J. Hazard. Mater..

[46] Chiou M.S., Li H.Y. (2002). Equilibrium and kinetic modeling of adsorption of reactive dye on cross-linked chitosan beads. J. Hazard. Mater..

[47] Dubinin M.M. (1960). The potential theory of adsorption of gases and vapors for adsorbents with energetically non-uniform surface. Chem. Rev..

[48] Kousha M., Daneshvar E., Salar-Sohrabi M., Jokar M., Bhatnagar A. (2012). Adsorption of acid orange II dye by raw and chemically modified brown macroalga Stoechospermum marginatum. Chem. Eng. J..

[49] Ho Y.S., McKay G. (1998). Sorption of dye from aqueous solution by peat. Chem. Eng. J..

[50] Silva J.P., Sousa S., Rodrigues J., Antunes H., Porter J.J., Gonçalves I., Ferreira-Dias S. (2004). Adsorption of acid orange 7 dye in aqueous solutions by spent brewery grains. Sep. Purif. Technol..

[51] Ho Y.S., McKay G. (1999). Pseudo-second order model for sorption processes. Process Biochem..

[52] Kara M., Yuzer H., Sabah E., Celik M.S. (2003). Adsorption of cobalt from aqueous solutions onto sepiolite. Water Res..

[53] Weber W.J., Morris J.C. (1963). Kinetics of adsorption on carbon from solution. J. Sanit. Eng. Div..

[54] Petit C., Burress J., Bandosz T.J. (2011). The synthesis and characterization of copper- based metal-organic framework/graphite oxide composites. Carbon.

