# Supplementary material for: Adsorption of Azo-Dye Orange II from Aqueous Solutions Using a Metal-Organic Framework Material: Iron- Benzenetricarboxylate
Source: Materials (Basel). 2014 Dec 12;7(12):8037–57. doi: 10.3390/ma7128037 (PMC5456435; doi:10.3390/ma7128037)
Supplement: Supplementary file 1 [file materials-07-08037-s001.pdf]

Supplementary Materials

Figure S1. Kinetic models plots for the adsorption of Orange II on Fe-BTC at different initial concentrations, (**a**) pseudo-second-order kinetic model plot; (**b**) pseudo-first-order kinetic model plot. (adsorbent mass: 10 mg; temperature: 298 K; pH 7).

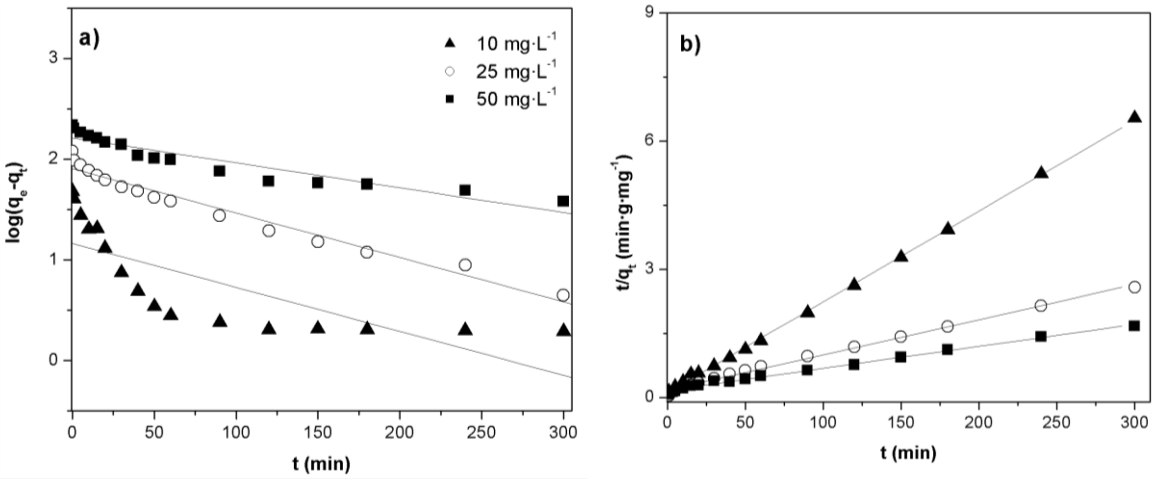

Figure S2. Kinetic models plots for the adsorption of Orange II on Fe-BTC at different initial concentrations, (**a**) pseudo-second-order kinetic model plot; (**b**) pseudo-first-order kinetic model plot. (adsorbent mass: 10 mg; temperature: 318 K; pH 7).

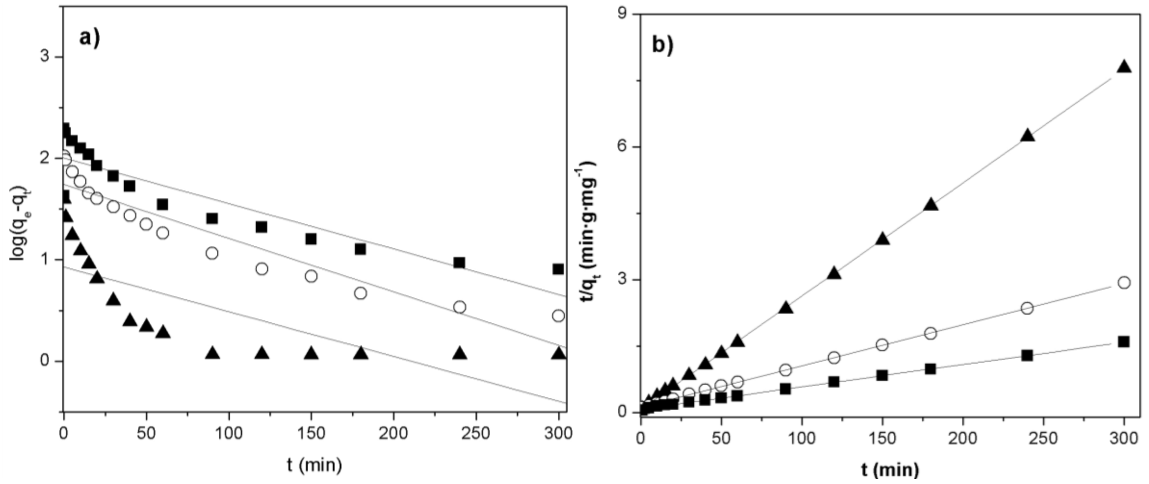

Figure S3. Intra-particle diffusion model plot of dye adsorbed on Fe(BTC). (Initial concentration: 50 mg·L^{-1}; adsorbent mass: 10 mg; temperature: 298 K and pH 7.

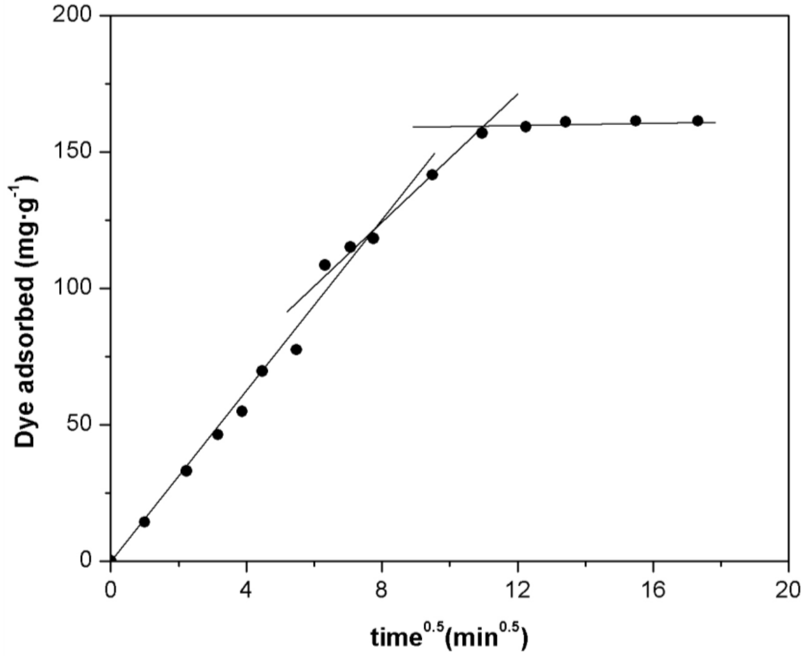

Figure S4. Intra-particle diffusion model plot of dye adsorbed on Fe(BTC). (Initial concentration: 50 mg·L^{-1}; adsorbent mass: 10 mg; temperature: 318 K and pH 7).

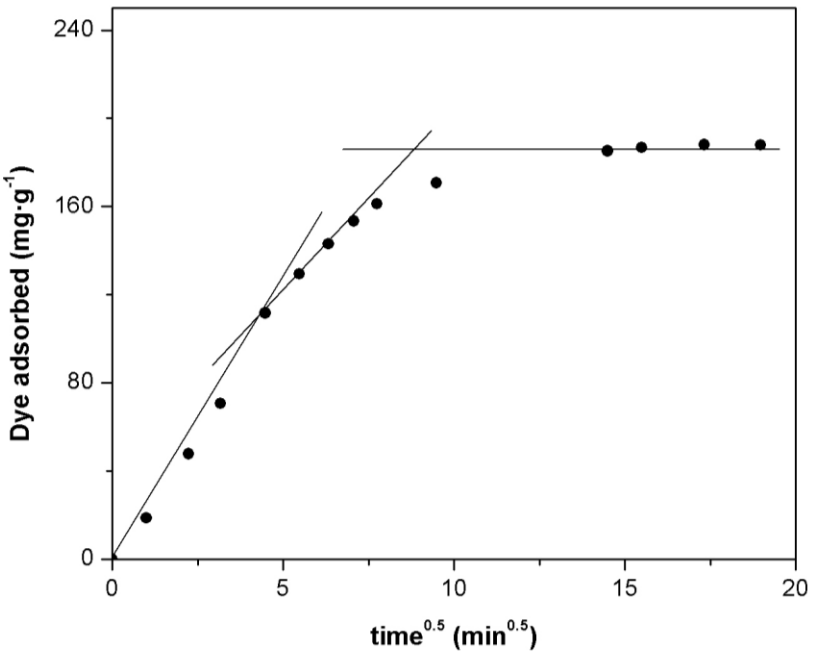

Table S1. Adsorbed amount (mg·g^{-1}) in the adsorption of Orange II dye over Fe(BTC) and activated carbon at different initial dye concentrations.

**Adsorbent**	**Adsorbed amount (mg·g^{-1})**		
	**10 ppm**	**25 ppm**	**50 ppm**
Fe(BTC)	43.98	115.49	212.49
Activated carbon	41.43	61.88	80.31

Table S2. Kinetic parameters for the adsorption of Orange II over Fe(BTC) at different concentrations and temperature.

**T**	**C_0**	**$q_{e,exp}$**	**Pseudo-first-order**			**Pseudo-second-order**		
**(°C)**	**(mg·g^{-1})**	**(mg·g^{-1})**	**$q_{e,cal}$ (mg·g^{-1})**	**k_1 (g^{-1}·mg·min)**	**R^2**	**$q_{e,cal}$ (mg·g^{-1})**	**k_2 (g^{-1}·mg·min)**	**R^2**
25	10	45.78	21.69	2.21×10^{-2}	0.762	47.85	3.61×10^{-3}	0.997
	25	116.02	80.50	1.01×10^{-2}	0.967	120.48	4.36×10^{-4}	0.992
	50	213.24	132.25	5.75×10^{-3}	0.940	217.39	1.09×10^{-4}	0.997
35	10	43.96	10.67	1.24×10^{-2}	0.617	44.8	4.14×10^{-3}	0.999
	25	115.49	45.91	1.31×10^{-2}	0.820	117.1	1.46×10^{-3}	0.999
	50	212.49	91.94	1.08×10^{-2}	0.750	220.3	5.64×10^{-4}	0.999
45	10	38.51	39.68	3.79×10^{-2}	0.862	39.68	8.35×10^{-3}	0.998
	25	104.10	53.48	5.52×10^{-3}	0.512	105.26	1.69×10^{-3}	0.998
	50	187.90	101.74	8.75×10^{-3}	0.853	196.08	1.03×10^{-3}	0.999
